# Ultrasound-Mediated Drug Delivery With a Clinical Ultrasound System: *In Vitro* Evaluation

**DOI:** 10.3389/fphar.2021.768436

**Published:** 2021-10-19

**Authors:** Josanne S. de Maar, Charis Rousou, Benjamin van Elburg, Hendrik J. Vos, Guillaume P.R. Lajoinie, Clemens Bos, Chrit T.W. Moonen, Roel Deckers

**Affiliations:** ^1^ Imaging and Oncology Division, University Medical Center Utrecht, Utrecht University, Utrecht, Netherlands; ^2^ Department of Pharmaceutical Sciences, Utrecht Institute for Pharmaceutical Sciences (UIPS), Utrecht University, Utrecht, Netherlands; ^3^ Physics of Fluids Group, MIRA Institute of Biomedical Technology and Technical Medicine, University of Twente, Enschede, Netherlands; ^4^ Laboratory of Acoustical Wavefield Imaging, Faculty of Applied Sciences, Delft University of Technology, Delft, Netherlands

**Keywords:** USMB, sonoporation, sonopermeation, ultrasound, microbubbles, chemotherapy, drug delivery, imaging

## Abstract

Chemotherapy efficacy is often reduced by insufficient drug uptake in tumor cells. The combination of ultrasound and microbubbles (USMB) has been shown to improve drug delivery and to enhance the efficacy of several drugs *in vitro* and *in vivo*, through effects collectively known as sonopermeation. However, clinical translation of USMB therapy is hampered by the large variety of (non-clinical) US set-ups and US parameters that are used in these studies, which are not easily translated to clinical practice. In order to facilitate clinical translation, the aim of this study was to prove that USMB therapy using a clinical ultrasound system (Philips iU22) in combination with clinically approved microbubbles (SonoVue) leads to efficient *in vitro* sonopermeation. To this end, we measured the efficacy of USMB therapy for different US probes (S5-1, C5-1 and C9-4) and US parameters in FaDu cells. The US probe with the lowest central frequency (i.e. 1.6 MHz for S5-1) showed the highest USMB-induced intracellular uptake of the fluorescent dye SYTOX™ Green (SG). These SG uptake levels were comparable to or even higher than those obtained with a custom-built US system with optimized US parameters. Moreover, USMB therapy with both the clinical and the custom-built US system increased the cytotoxicity of the hydrophilic drug bleomycin. Our results demonstrate that a clinical US system can be used to perform USMB therapy as efficiently as a single-element transducer set-up with optimized US parameters. Therefore, future trials could be based on these clinical US systems, including validated US parameters, in order to accelerate successful translation of USMB therapy.

## Introduction

Chemotherapy is typically used as systemic treatment to destroy metastatic cancer cells that have spread away from the primary tumor. However, local action of chemotherapy is also of importance throughout the spectrum of oncological therapy. First, surgically unresectable tumors can be made operable with neoadjuvant chemotherapy (Sclafani et al., 2017; Fietkau et al., 2021). Secondly, neoadjuvant chemotherapy can lead to less extensive surgery and reduce the risk of local recurrences (Dietz et al., 2018; van Ramshorst et al., 2018). Third, chemotherapy can enhance the local effect of radiotherapy during chemoradiation (Geoffrois et al., 2018; Versteijne et al., 2020; Zhao et al., 2020). Finally, local response to palliative chemotherapy can decrease morbidity (Vermorken et al., 2008; Judson et al., 2014; Loupakis et al., 2014). Unfortunately, there is substantial heterogeneity in the local response to systemic treatment within and across cancer types. A plausible explanation for suboptimal response is the heterogeneous and/or insufficient delivery of drugs to tumor cells caused by biophysical barriers of the tumor tissue (Tredan et al., 2007; de Maar et al., 2020).

The combination of ultrasound and microbubbles (USMB) has been shown to overcome these biophysical barriers and increase local tumor uptake of several drugs resulting in enhanced efficacy (Lammertink et al., 2015b; Snipstad et al., 2018; Kooiman et al., 2020). Microbubbles are micron sized (1–10 µm in diameter) gas-filled particles with a biocompatible shell that are widely used as vascular contrast agents for ultrasound imaging (Chong et al., 2018; Frinking et al., 2020). In addition, the interaction of US waves and microbubbles has the potential to enhance the delivery of drugs. Microbubbles exposed to low-intensity US fields will oscillate (i.e., stable cavitation), while microbubbles exposed to higher intensities will collapse violently (i.e., inertial cavitation). Both types of cavitation lead to a number of bio-effects collectively known as sonopermeation, such as the formation of pores in cell membranes (sonoporation), enhanced endocytosis and increased vascular permeability, that improve the deposition of drugs in tumor tissue (Snipstad et al., 2018; Deprez et al., 2021).

In particular, hydrophilic drugs such as bleomycin and cisplatin, that have difficulties crossing the cell membrane, may benefit from local USMB therapy, leading to increased delivery of such drugs *in vitro* (Iwanaga et al., 2007; Watanabe et al., 2008; Maeda et al., 2009; Heath et al., 2012; Sasaki et al., 2012; Lamanauskas et al., 2013; Sato et al., 2014; Sato et al., 2015; Lammertink et al., 2016; Tamosiunas et al., 2016; Hirabayashi et al., 2017; Sasaki et al., 2017; Chen et al., 2018; Fekri et al., 2019) and increased anti-tumor response *in vivo* (Iwanaga et al., 2007; Watanabe et al., 2008; Heath et al., 2012; Sato et al., 2014; Sato et al., 2015; Hirabayashi et al., 2017; Chen et al., 2018). The first clinical trials using the combination of chemotherapy and USMB have been conducted. In a phase 1 clinical trial, USMB with clinically available microbubble SonoVue was combined with gemcitabine in ten inoperable pancreatic cancer patients. Trial participants could tolerate significantly more treatment cycles and the median overall survival was longer compared to historical controls treated with gemcitabine alone (Kotopoulis et al., 2013; Dimcevski et al., 2016). Another phase 1 trial in eleven patients with hepatic metastases and one patient with pancreatic cancer concluded that treatment with physician’s choice chemotherapy (most commonly FOLFIRI, i.e., folinic acid, fluorouracil and irinotecan) plus USMB with SonoVue was safe (Wang et al., 2018). Several follow-up phase 1/2 studies are currently recruiting or being prepared (Clinicaltrials.gov NCT04146441, NCT04821284, NCT03477019 and NCT03458975). A phase 3 trial investigates the addition of USMB to neoadjuvant chemotherapy in breast cancer (Clinicaltrials.gov NCT03385200, current status unknown).

Despite these promising developments, translation of USMB therapy from *in vitro* and small animal studies to the clinic is still limited. One major obstacle for clinical translation of USMB therapy is the lack of a clinically approved US system with settings optimized to perform USMB therapy. A large variety of (non-clinical) US set-ups and US parameters have been used for *in vitro* and *in vivo* studies (Roovers et al., 2019). While these studies have provided invaluable insights on the underlying mechanisms of USMB therapy and provided *in vivo* proof of concept, their methods and results cannot be easily transferred to clinical studies because the US equipment is not, and will not likely be, approved for clinical use. In this study we take a different approach to facilitate the clinical translation of USMB therapy by investigating the potential of an existing clinical ultrasound system (Philips iU22) in combination with clinically available microbubbles (SonoVue) to perform USMB therapy. To this end, we use *in vitro* experiments to evaluate the effect of USMB therapy on the intracellular uptake of a model drug (SYTOX™ Green) and the efficacy of the hydrophilic chemotherapeutic drug bleomycin.

## Materials and Methods

### Chemicals and Reagents

All cells were cultured in high glucose Dulbecco’s modified eagle medium (DMEM) supplemented with 10% fetal bovine serum (FBS) and 1% non-essential amino acids (NEAA).

Dulbecco’s Phosphate Buffered Saline (PBS), modified, without calcium, chloride and magnesium chloride was used as solvent and for washing steps.

PBS, DMEM, FBS, NEAA and trypsin/ethylenediaminetetraacetic acid (EDTA) were purchased from Sigma-Aldrich (St. Louis, United States).

Bleomycin sulfate (Bleomedac® powder for solution for injection, GmbH, Wedel, Germany) was dissolved in sterile 0.9% NaCl to reach a final concentration of 10 μg/ml, which corresponds to at least 15IU (Ph. Eur) per ml. Bleomycin is hydrophilic (LogP-7.5) and has a molecular weight of 1,415.6 g/mol^1^.

SYTOX™ Green is a cell-impermeant fluorescent nuclear acid stain with excitation/emission wavelength of 504/523 nm. Its impermeability and >500-fold fluorescence enhancement after binding to nuclear acids makes it suitable to visualize USMB therapy efficacy. DRAQ5™ fluorescent probe is a cell-permeant fluorescent dye (excitation 647 nm, emission 681 nm) that was used to counterstain the DNA content of all cells. AlamarBlue™ reagent was used for the cell viability assay. The eBioscience™ Annexin V Apoptosis Detection Kit APC, containing both fluorescently labelled Annexin V and Propidium Iodide (PI), was used for the apoptosis assay. SYTOX™ Green, DRAQ5™, AlamarBlue™ and eBioscience™ Annexin V Apoptosis Detection Kit APC were purchased from ThermoFisher Scientific (Waltham, Massachusetts, United States).

### Cell Culture

A human pharyngeal squamous cell carcinoma cell-line (FaDu) (ATCC® HTB-43™, LGC Standards GmbH, Wedel, Germany) was cultured in high glucose DMEM supplemented with 10% FBS and 1% NEAA. FaDu cells were cultured in a humidified incubator at 37°C and 5% CO_2_. They were split 2–3 times per week at a confluency of around 80%, until a maximum passage number of 20. One day before each experiment, FaDu cells collected using trypsin/EDTA and seeded in a 35 mm diameter lumox® culture dish (Sarstedt AG & Co. KG, Nümbrecht, Germany).

### Ultrasound Systems and Microbubbles

SonoVue (Bracco International B.V., Amsterdam, Netherlands) was prepared according to the manufacturer’s instructions, producing sulfurhexafluoride-filled phospholipid microbubbles with a mean bubble diameter of ∼2.5 µm and a concentration of 1–5*10^8^ microbubbles/ml in sterile 0.9% NaCl. Microbubbles were kept at 4°C in between use, resuspended before every use and used within 2 h after preparation.

We used a clinical ultrasound system (iU22 Ultrasound system, Philips Medical Systems Nederland B.V., Best, Netherlands) combined with the following probes: S5-1, C5-1 and C9-4. USMB therapy was done in Pulsed Wave (PW) Doppler mode. The transmission frequency of each transducer was set by the system and cannot be changed. The Pulse Repetition Period (PRP) was set to the longest period for each transducer by setting the scale parameter to the minimum. The acoustic pressure was varied by changing the mechanical index (MI). The number of cycles per pulse was varied by changing the sample volume (SV), while the MI (pressure) was kept constant.

The acoustic field of each transducer in PW Doppler mode as well as the acoustic pressure for each setting was measured using a 0.2 mm needle hydrophone (Precision acoustics Ltd., Dorset, United Kingdom) in degassed water.

As reference, we used a custom-built US set-up that was previously used for USMB therapy (Lammertink et al., 2015a). This US set-up consisted of a single-element transducer operated at 1.5 MHz, 150 cycles per pulse, pulse repetition frequency of 1.0 kHz and Peak negative pressures (P_neg_) of 0.39, 0.56 and 0.72 MPa.

### Microbubble Response

The acoustic bubble response to the specific acoustic pulses used in the experiment was characterized by attenuation measurements. A sample holder (acoustical path length of 8 mm) with two acoustically transparent windows was positioned such that its center coincided with the focal point of two single-element transducers. The transmit transducer (Olympus V304, f = 2.25 MHz, F = 1.88 inch, D = 1 inch) was calibrated using a fibre-optic hydrophone (Precision Acoustics). The receiving transducer (Olympus V307, f = 5 MHz, F = 1.93 inch, D = 1 inch, was aligned such that the received signal (without microbubbles) was at a maximum.

Eight differently shaped US pulses were used, four with a rectangular envelop (as used in the single-element set-up) and four with a Gaussian envelop (to recreate the pulses of the US imager probe), with 11, 23, 46 and 150 cycles. These pulses were generated by a waveform generator (Tabor 8026) and amplified (vectawave, VBA100-200) before transmission. The receiving transducer and the waveform generator were connected to a digital oscilloscope (picoscope 5444d) such that both the transmitted and received signal were recorded. The waveform generator as well as the oscilloscope were triggered (BNC, 575) simultaneously.

Each US pulse was repeated 5 times, with a pulse repetition frequency (PRF) of 6.7 kHz, for five different Peak negative pressures (150–750 kPa with 150 kPa steps), such that one measurement consisted of 200 pulses. During the measurements the sample in the holder was continuously refreshed by a gravity-driven flow. Measurements were done at the frequencies used throughout the rest of the paper, namely 1.6, 2.25, and 4 MHz. All measurements were performed with diluted (1,000x) SonoVue and without microbubbles for reference.

Attenuation coefficients were calculated by comparing the transmission through the SonoVue solution to that through distilled water:

$$\alpha =-\frac{10}{d}\log_{10}\left(\frac{\;{\left|V_{S}\left(f_{t}\right)\right|}^{2}}{{\left|V_{R}\left(f_{t}\right)\right|}^{2}}\right),$$

where α is the attenuation coefficient in dB/cm, d is the acoustical path length through the sample in cm, $\left|V_{S}\left(f_{t}\right)\right|$ and $\left|V_{R}\left(f_{t}\right)\right|$ are the amplitudes of the frequency spectra of the SonoVue and reference signal, respectively, at the transmit frequency $f_{t}$ .

### USMB Therapy Experimental Set-Up

**FIGURE 1:**
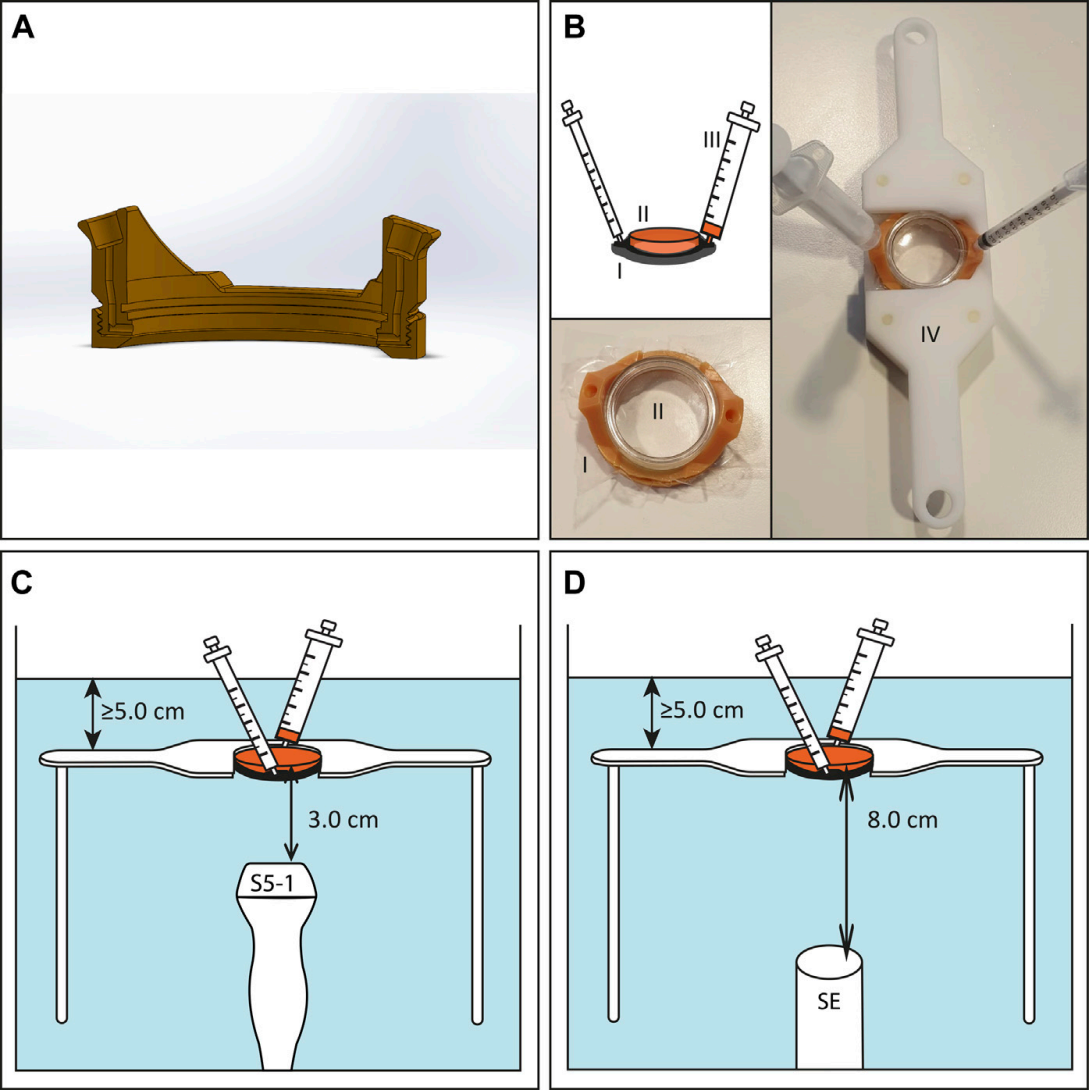
Experimental US set-up for *in vitro* experiments. **(A)** Cross section of 3D-printed TwentiCell **(B)** Schematic drawing and photos of TwentiCell (I) including upside-down inserted lumox^®^ dish (II) in custom-build frame (IV). A 5 ml syringe (III) is used to insert the medium and to remove air. **(C)** TwentiCell immersed in degassed water, in a custom-build frame, with clinical US probe 3.0 cm below the cells or **(D)** with single element transducer 8.0 cm below the cells. In both set-ups at least 5 cm of water was above the cells.

In order to apply USMB therapy to cells cultured in lumox® dishes we used TwentiCells, which were designed and manufactured at Twente University (Figure 1). The TwentiCell consists of a 3D-printed lumox® dish holder and a screw-on ring to seal the lumox® dish with a polyolefin (25 µm thick), creating a water-tight compartment. The holder contains an in-let and out-let to the fill the compartment with drugs/microbubbles in solution and remove unwanted air, respectively. The parts were assembled before each experiment and UV-sterilized before inserting the lumox® dish containing the cells to avoid infection. Acoustic transparency of the TwentiCells and inserted lumox® dishes is close to 100% (data not shown).

For the USMB therapy the TwentiCell was immersed in degassed water (T = 37°C) and fixed above the transducer in a custom-built frame. The distance between the surface of the clinical ultrasound probes and the lumox® dish membrane was 3.0 cm and the PW Doppler Sample Volume was centered on this position **(** Figure 1C **)**. In the single-element transducer set-up the lumox® dish membrane was positioned in the focal zone of the transducer, i.e. 8.0 cm above the transducer surface (Figure 1D).

### SYTOX™ Green Experiments

One day after seeding 3.0 × 10^5^ FaDu cells in lumox® dishes, the medium was removed and a mixture of 2 µl SYTOX™ Green (SG, 5 mM solution in DMSO), 180 µl SonoVue and 4.8 ml medium was added to the TwentiCell. For USMB-untreated samples, a lumox® dish with regular lid was used and the volumes were adjusted to 0.5 µl SG, 45 µl 0.9% NaCl and 1.2 ml medium. The TwentiCells were incubated (37°C and 5% CO_2_) for 15 min with the cell containing surface upwards, in order for the microbubbles to float towards the cells. Next, the TwentiCell was placed in the waterbath (cell containing surface remaining upwards), exposed to ultrasound for 15 s and put back in the incubator. The US-untreated samples were not removed from the incubator. 30 min after USMB treatment, the SG-containing medium was removed and clean medium was added. Afterwards, the cells were washed with PBS, fixated with paraformaldehyde (4% in PBS) and stained with 1 ml DRAQ5 (5 μM in PBS) at 37°C for about 20 min. The lumox® membrane was covered with solidifying mounting medium FluorSave™ (Merck Millipore, Burlington, Massachusetts, United States) and a glass cover slip, and kept in the dark at 4°C until fluorescence imaging. These experiments were performed with the single element transducer as well as the clinical US system.

### Bleomycin Experiments

One day after seeding 1.5 × 10^5^ FaDu cells in lumox® dishes, the medium was removed and a mixture of 498 µl bleomycin solution or 0.9% NaCl, 180 µl SonoVue and 4.302 ml medium was added to the TwentiCell. For US-untreated samples, a lumox® dish with regular lid was used and the volumes were adjusted to 125 µl bleomycin solution or 0.9% NaCl, 45 µL 0.9% NaCl and 1.075 ml medium. The TwentiCells were incubated (37°C and 5% CO_2_) for 15 min with the cell containing surface upwards. Next, the TwentiCell was placed in the waterbath (cell containing surface remaining upwards), exposed to ultrasound for 15 s and put back in the incubator. The US-untreated samples were not removed from the incubator.

To determine the IC_50_ and IC_25_ of bleomycin with or without USMB, the final bleomycin concentrations were 0, 0.1, 0.5, 1, 5, 10, 50, 100 and 500 μg/ml and the single-element transducer set-up was used. The apoptosis assay was performed at a single bleomycin concentration (10 μg/ml) using the single-element set-up as well as the clinical US system.

Two hours after USMB therapy, the bleomycin or 0.9% NaCl containing medium was removed, the cells were washed with PBS and clean medium was added. The cells were then incubated at 37°C, 5% CO_2_ until 48 h after adding the bleomycin, microbubbles and/or 0.9% NaCl.

### Fluorescence Microscopy

Fluorescence imaging for the SG experiments was performed on a Confocal Zeiss LSM 700 microscope. SG was imaged with excitation 488 nm and emission >500 nm. DRAQ5 was imaged with excitation 639 nm and emission >640 nm. All microscope settings, including laser power, gain, pinhole size and digital offset, were kept constant during all experiments. Images were obtained with 10 times enlargement with a frame size of 512 × 512 and a square tile size of 640.17 µm^2^. For each lumox® dish, a square of 10 by 10 tiles was imaged with a 10% overlap, starting in the visual center of the SG signal. The tiles were stitched immediately after acquisition. In each tile a Z-stack of three levels was created to compensate for height variances of the cells over the tiles.

To quantify the USMB efficacy for different US-settings we performed automated cell segmentation of SG-positive and DRAQ5-positive cells using (Fiji Is Just) ImageJ 2.0.0-rc-69. First, a standard-deviation Z-projection was created for the SG and DRAQ5 images. To segment the SG-positive cells global thresholding was applied, with a fixed threshold for all samples, whereas for segmenting the DRAQ5-positive cells a local threshold was applied (i.e., mean method with a radius of 5). Next, the noise in the binary masks after thresholding was removed with a median filter and the watershed algorithm was applied to split clustered objects. Objects with a size ≥20 pixel units were counted as cells, regardless of circularity.

The number of SG and DRAQ5-positive cells were analyzed in a region of interest (ROI) of 600 × 600 pixels, centered on the position with the highest SG signal after blurring the SG image with a 2-D Gaussian smoothing kernel with standard deviation of 200 in Matlab (R2019a). When there was no noteworthy SG signal, the ROI was positioned in center of the 10 × 10 square. Objects on the edges of the ROI were not counted.

### Viability Assay

The effect of bleomycin with or without USMB therapy on cell viability was determined with an AlamarBlue assay. 48 h after adding medium with or without bleomycin and microbubbles to the Twenticell, a solution of 1 ml medium and 100µ AlamarBlue reagent (500 µM solution in PBS) was added to each lumox® and incubated (37°C and 5% CO_2_). After 2 h, the mixture was removed from each lumox® and pipetted into a well plate.

The fluorescence intensity in the well plate was measured using the FLUOstar OPTIMA (BMG LABTECH) plate reader, with excitation and emission wavelengths of 550–10 and 600–610 nm and a gain of 1,500. The cell viability of a sample was calculated as percentage fluorescent signal relative to that of untreated control samples, after subtraction of the fluorescent signal of a negative control without cells.

To determine IC_50_/IC_25_ in each group (with or without USMB), the cell viability percentages were calculated with reference to their own controls, i.e., no exposure to bleomycin but with or without USMB depending on the group. The IC_50_ was then defined as the concentration resulting in 50% inhibition of cell viability, likewise, the IC_25_ was the concentration resulting in 25% inhibition of cell viability. The method to determine IC_50_/IC_25_ is described in *Statistical Analysis*.

### Apoptosis Assay

In addition to the viability assay, an apoptosis assay was performed to determine the effect of USMB on bleomycin efficacy. The apoptosis and viability assays were performed in separate experiments. 48 h after adding medium with or without bleomycin and with or without microbubbles to the Twenticell, the medium and detached cells were collected from each lumox® dish. The remaining cells were detached from the lumox® membrane with trypsin/EDTA and added to the rest of the medium. Residual EDTA was removed by centrifugation and washing with PBS. The cells were resuspended in binding buffer with a concentration of ∼1 × 10^6^ cells/ml and then stained and incubated for 15 min with Annexin V. The cells were washed, resuspended in binding buffer, stained with Propidium Iodide (PI) and then kept on ice protected from light.

Within 4 h, the samples were analyzed by flow cytometry using the BD FACSCanto ™ II Cell Analyzer, for PI (488 nm) and Annexin V (633 nm). Compensation was performed with samples stained with only PI and only Annexin V. The FACS data was analyzed using FlowJo 10.7.1. The four quadrants (live, early apoptotic, late apoptotic, and necrotic cells) were distinguished based on a control sample containing 50% necrotic and 50% live cells.

### Statistical Analysis

Statistical analysis was performed in GraphPad Prism 8.3.0. For the fluorescence microscopy data and cell viability data we used the Kruskal Wallis test and a Dunn’s test for multiple comparisons. The absolute IC_50_ for bleomycin with and without USMB was determined with a nonlinear least-squares regression of the bleomycin concentration versus the response (cell viability percentage) with the Hill’s slope fixed at −1.0 and the top and bottom of the fitted curve restrained to 100 and 0%, respectively. To compare the IC_50_’s of both groups we used the extra sum of squares F-test. Because the IC_50_’s had a very broad confidence interval we also calculated the IC_25_ for both groups with the same method. A *p*-value < 0.05 was considered to indicate a statistically significant difference.

## Results

### Acoustic Characterization of Clinical US System

**FIGURE 2:**
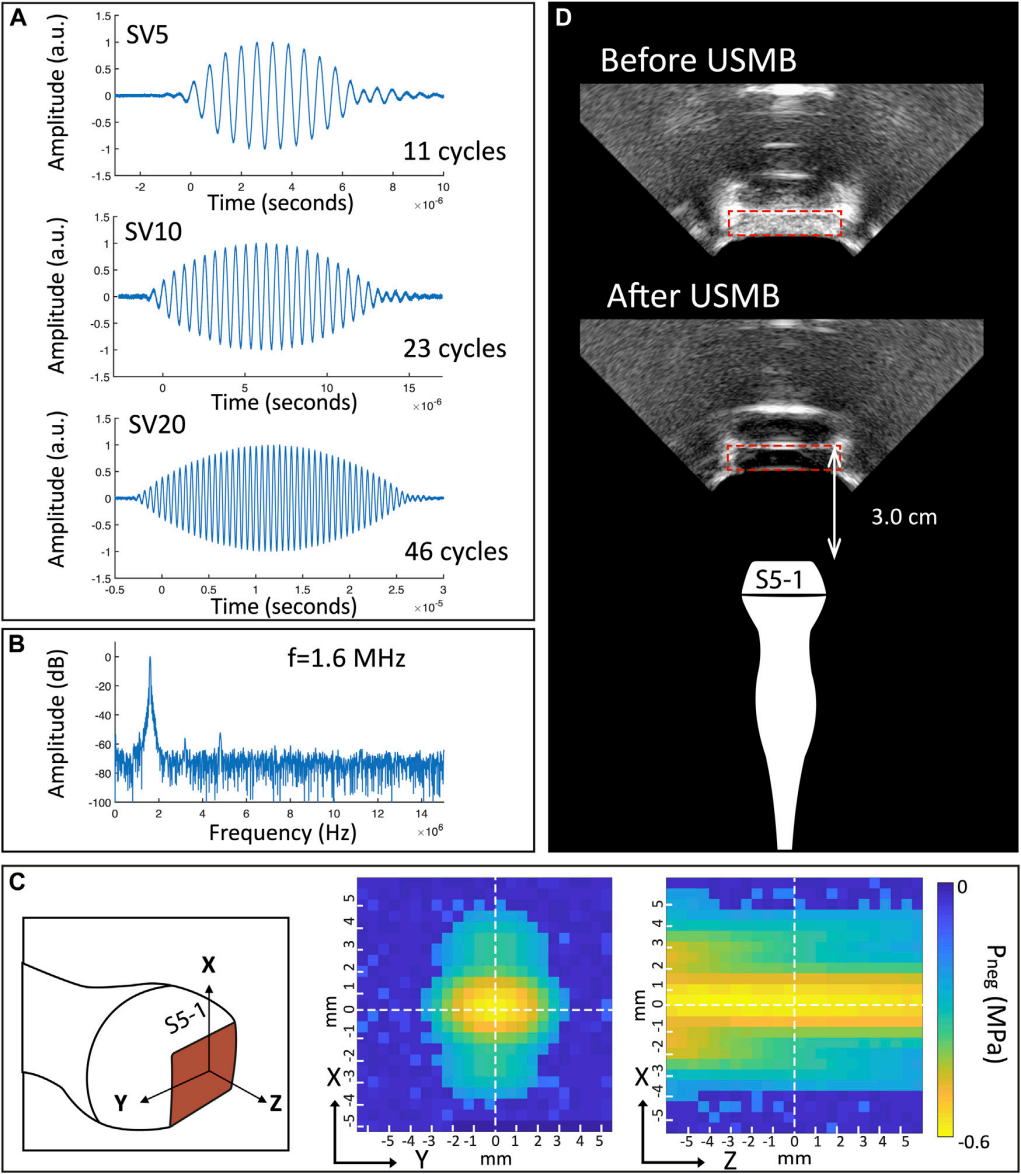
Characteristics of S5-1 probe **(A)** Shape of emitted ultrasound pulses in PW mode for different pulse length. **(B)** Frequency spectrum of SV 20 mm pulse. **(C)** Pressure field maps in PW mode. **(D)** B-mode images of TwentiCell (red rectangle) containing microbubbles, before and after USMB therapy (15 s at MI 0.6, SV 20 mm). SV: sample volume; f: frequency; a.u.: arbitrary units; P_neg_: Peak negative pressure; USMB: ultrasound and microbubbles.

The acoustic output of the clinical ultrasound system as well as the US beam profile were characterized for the probes S5-1, C5-1 and C9-4. The acoustic output as function of different US settings is summarized in Table 1. With increasing SV, the number of cycles per pulse increased. The maximum MI (and therefore pressure) increased with decreasing SV. Figure 2 shows the characteristics of the S5-1 probe. Supplementary Figures S1–S3 show these characteristics for the other clinical US probes and the single-element transducer. In PW mode the S5-1 probe emits a US pulse with a Gaussian shaped envelope with a center frequency of 1.6 MHz and an increasing number of cycles when SV is increased (Figures 2A,B). The pressure field maps in PW mode **(** Figure 2C **)** show that the ultrasound energy is limited to a beam with dimensions of 5.0 mm by 6.3 mm (Full width at half maximum) at the middle of the sample volume. Figure 2D demonstrates the difference in signal intensity in the Twenticell (red rectangle) before and after USMB therapy (15 s sonication at MI 0.6, SV 20 mm), due to microbubble disruption.

**TABLE 1:** US parameters and corresponding measurements on clinical US system.

Clinical US probe		Pulse repetition period		Pulse length		Maximum pressure at SV 20 mm		Evaluated pressures atSV 20 mm	
	PW freq (MHz)	Min. Scale (cm/sec)	Max. PRP (µs)	SV (mm)	Cycles per pulse	Max. MI	Max. P_neg_ (MPa)	MI evaluated	P_neg_ (MPa)
S5-1	1.6	−30 to 30	800	20	46	0.6	0.59	0.6	0.59
				10	23			0.5	0.46
				5	11			0.4	0.38
								0.3	0.30
								0.2	0.22
C5-1	2.25	−6 to 6	2,500	20	64	0.8	0.59	0.8	0.59
				10	32			0.5	0.39
				5	16			0.3	0.25
C9-4	4.0	−12 to 12	800	20	117	0.3	0.44	0.3	0.44
				10	59			0.2	0.37
				5	29			0.2	0.30
								0.1	0.19

### Bubble Response to US

**FIGURE 3:**
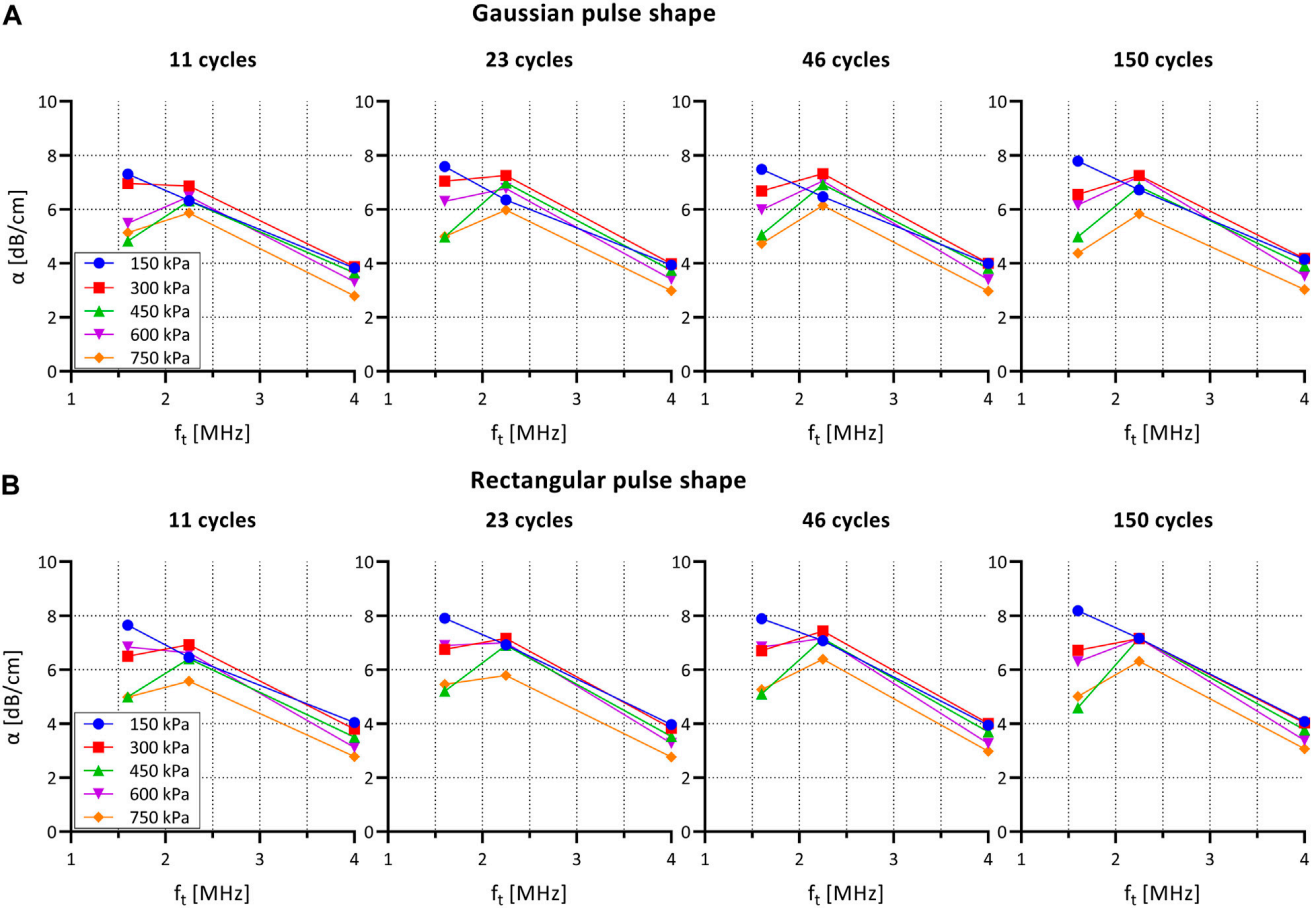
Attenuation coefficient α for SonoVue for different pulse shapes and lengths as a function of acoustic pressure and frequency. **(A)** Gaussian pulse envelop (as used in PW Doppler mode on clinical US system). **(B)** Rectangular pulse envelop (as used in single-element set-up). α: attenuation coefficient; f_t_: transmit frequency.

Figure 3 shows the attenuation coefficient for SonoVue for different pulse envelop shapes and lengths as a function of acoustic pressure and frequency.

The attenuation curve shows a maximum at 1.6 and 2.25 MHz indicating a maximum attenuation for the frequencies that are closest to resonance frequency of SonoVue.

The attenuation coefficient decreased, mainly at 1.6 MHz, as the excitation pressure was increased from 150 to 750 kPa. This observation is not consistent with previous experimental measurements of pressure-dependent attenuation coefficients (Tang and Eckersley, 2007; Emmer et al., 2009). As we have observed no visible trace of bubble destruction based on the repeated pulses, the refreshment rate of the bubble solution appears to be sufficient, and we ascribe this effect to radiation forces and bubble clustering. As such, this effect may be even more prominent at the higher concentrations used for the cell experiments.

When comparing rectangular versus Gaussian envelop shapes no differences were observed in attenuation coefficient. Furthermore, the number of cycles per pulse did not influence the attenuation coefficient.

### Effect of Transducer and Ultrasound Settings on USMB Efficiency

**FIGURE 4:**
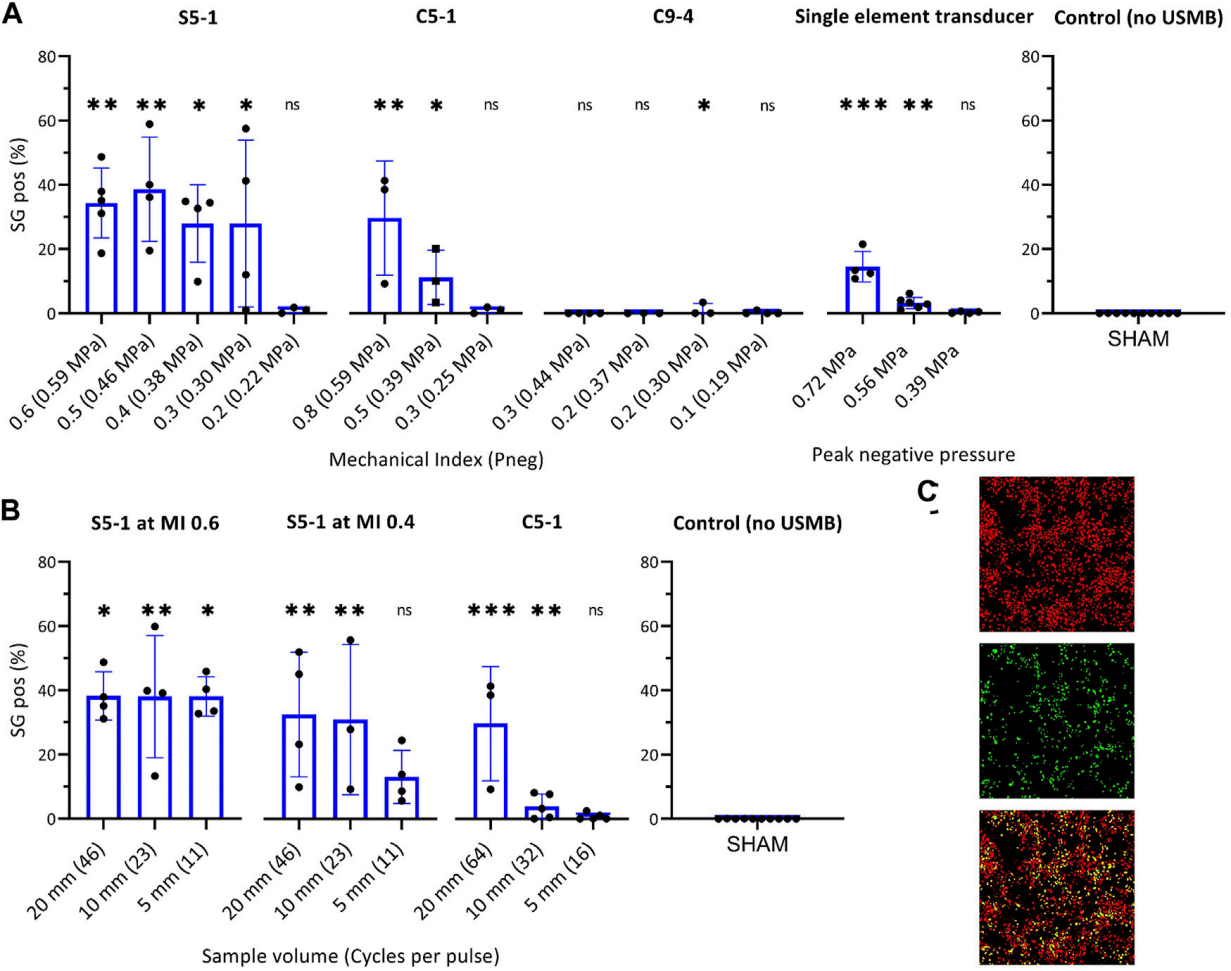
Effect of transducer and ultrasound settings on USMB efficiency measured by percentage SG positive cells in ROI. **(A)** Effect of pressure on USMB efficiency with (from left to right) clinical US system with S5-1, C5-1 or C9-4 probe and custom-build US set-up with single-element transducer. **(B)** Effect of pulse length on USMB efficiency of clinical US system with (from left to right) S5-1 probe at maximum pressure (MI 0.6, P_neg_ 0.59 MPa), S5-1 probe at MI 0.4 (P_neg_ 0.38 MPa) and C5-1 probe at maximum pressure (MI 0.8, P_neg_ 0.59 MPa). Symbols indicate individual measurements and bars indicate mean and SD (*n* ≥ 3) All values were statistically compared to USMB untreated samples (right). *p < 0.05 **p < 0.01 ***p < 0.001. **(C)** Representative fluorescence images of ROI after USMB with S5-1 probe at MI 0.6 (0.59 MPa) and maximum pulse length (SV 20 mm). Top: DRAQ5™ staining, middle: SG staining, Bottom: composite, cells stained for DRAQ5™ (red), SG (green) or both (yellow). SG uptake in 35.1% of cells. ns: not significant; SG: SYTOX™ Green; USMB: ultrasound and microbubbles.

The USMB efficacy for each transducer as function of acoustic pressure is shown in Figure 4A. The percentage of SG positive cells increased significantly with the addition of USMB treatment using the S5-1 (1.6 MHz) or C5-1 (2.25 MHz) probes and was comparable (C5-1) or even higher (S5-1) than with the single element transducer (Figure 4A). In contrast, no relevant SG uptake was observed using the C9-4 probe (4.0 MHz). For the S5-1 probe a similar percentage of SG positive cells (i.e., ∼30%) was observed for all pressures above 0.30 MPa. In contrast, the C5-1 probe and the single element transducer showed a pressure-dependent increase of SG positive cells, reaching ∼30 and 15% at the highest pressures, respectively.

The effect of pulse length for the S5-1 and C5-1 is shown in Figure 4B. For the S5-1 probe at maximum pressure (0.59 MPa), a similar percentage of SG positive cells (i.e., 38%) was observed independent of pulse length. For the S5-1 probe at a lower pressure (0.38 MPa) only a higher number of cycles per pulse (SV 20 and 10 mm) caused a significant increase in SG positive cells compared to USMB untreated samples. For the C5-1 probe at maximum pressure (0.59 MPa), the percentage SG positive cells increased with increasing pulse length.

Figure 4C shows representative fluorescence images of the ROI after USMB therapy with S5-1 probe at MI 0.6 and SV 20 mm, stained with SG and DRAQ5.

### Effect of USMB on Bleomycin Efficacy

**FIGURE 5:**
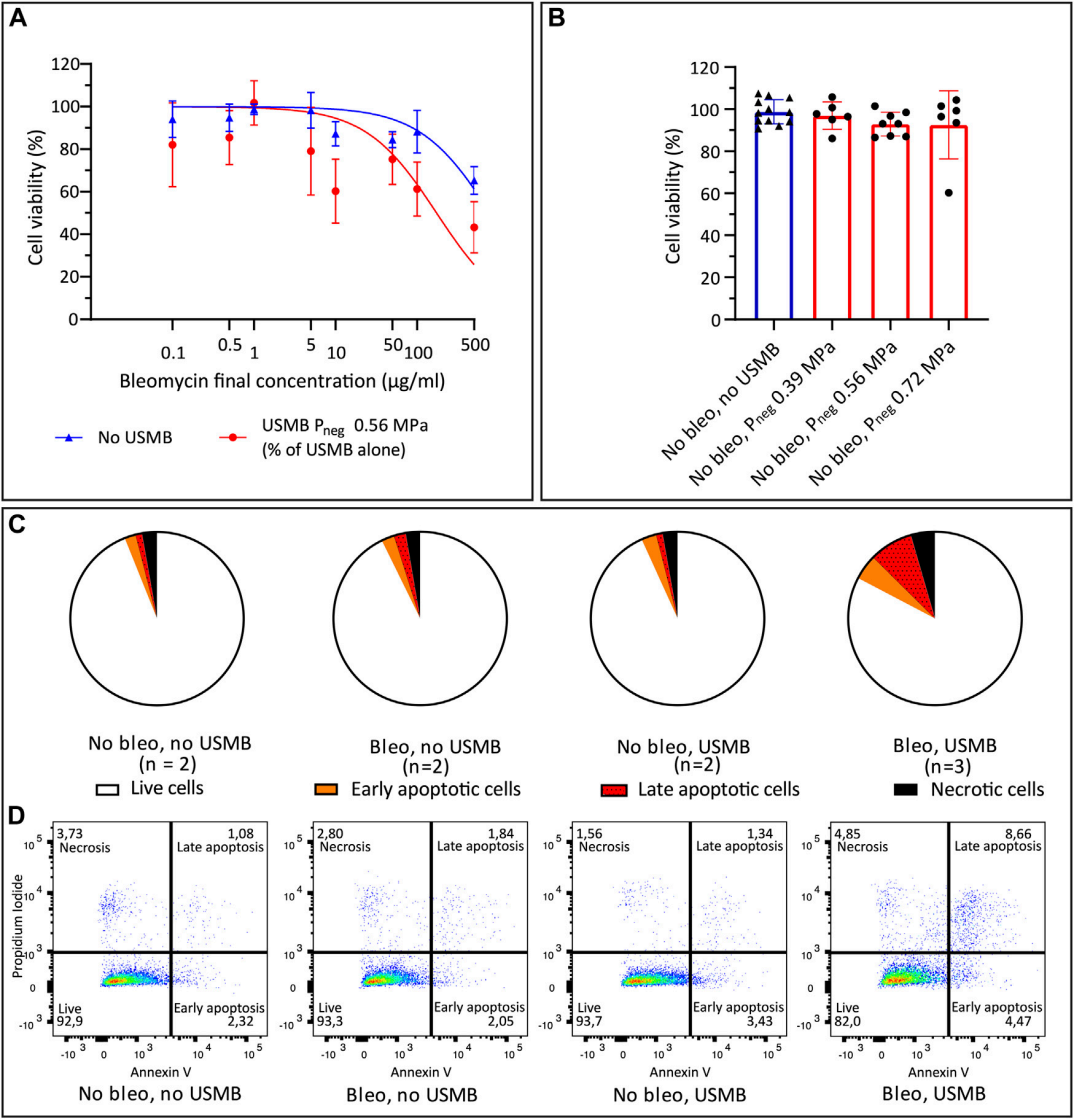
Cell viability after bleomycin ± USMB using the custom-build US set-up with single element transducer. **(A)** Nonlinear least-squares regression of bleomycin concentration versus cell viability percentage (relative to samples without bleomycin), with (red circles) and without (blue triangles) USMB at P_neg_ 0.56 MPa. Symbols and bars indicate mean and SD (*n* ≥ 3). **(B)** Cell viability with (red, circles) or without (blue, triangles) USMB alone at three pressures, symbols indicate individual measurements and bars indicate mean and SD (*n* ≥ 6). **(C)** Flow cytometry analysis of Propidium Iodide and Annexin V staining 48 h after bleomycin 10 μg/ml or NaCl 0.9% with or without USMB at P_neg_ 0.56 MPa. Pie charts represent the mean of the samples with *n* = 2 or (for bleomycin + USMB samples) *n* = 3. **(D)** Representative dot plots of flow cytometry analysis shown in **(C)**. More apoptosis was observed after bleomycin + USMB. Bleo: bleomycin; P_neg_: Peak negative pressure; USMB: ultrasound and microbubbles.

To confirm that USMB therapy improved the efficacy of bleomycin, the IC_50_ and IC_25_ of bleomycin were determined, with or without USMB with the single-element transducer **(** Figure 5A)**.** The absolute IC_50_ of bleomycin decreased from 791.8 μg/L (95% CI 578.8–1,125) to 173.2 μg/ml (95% CI 96.06–333.0) when combined with USMB (P_neg_ = 0.56 MPa). The USMB-induced difference in IC_50_ was statistically significant (*p* < 0.0001). The IC_25_ decreased significantly from 263.9 μg/ml (95% CI 192.9–374.8) to 57.73 μg/ml (95% CI 32.02–111.0) (*p* < 0.0001). Note that both curves have their own reference of 100% cell viability without bleomycin (i.e., with or without USMB), which guarantees that the observed differences in IC_25_ and IC_50_ are not a direct cytotoxic effect of USMB alone, but due to enhanced intracellular delivery of bleomycin causing more cell death at the same concentration. The effect of USMB treatment in the absence of bleomycin is illustrated in Figure 5B. Cell viability did not significantly decrease with increasing acoustic pressure.

The apoptosis assay confirmed the decreasing cell viability with addition of USMB to bleomycin. Figure 5C shows the mean distribution of cells over the quadrants after USMB with the single-element transducer. Representative dot plots of flow cytometry analysis from experiments with two or three samples per group are shown in Figure 5D. Increased apoptosis was observed 48 h after bleomycin plus USMB (P_neg_ 0.56 MPa), compared to untreated samples or samples treated with either bleomycin alone or USMB alone.

### Effect of USMB With Clinical US System on Bleomycin Efficacy

**FIGURE 6:**
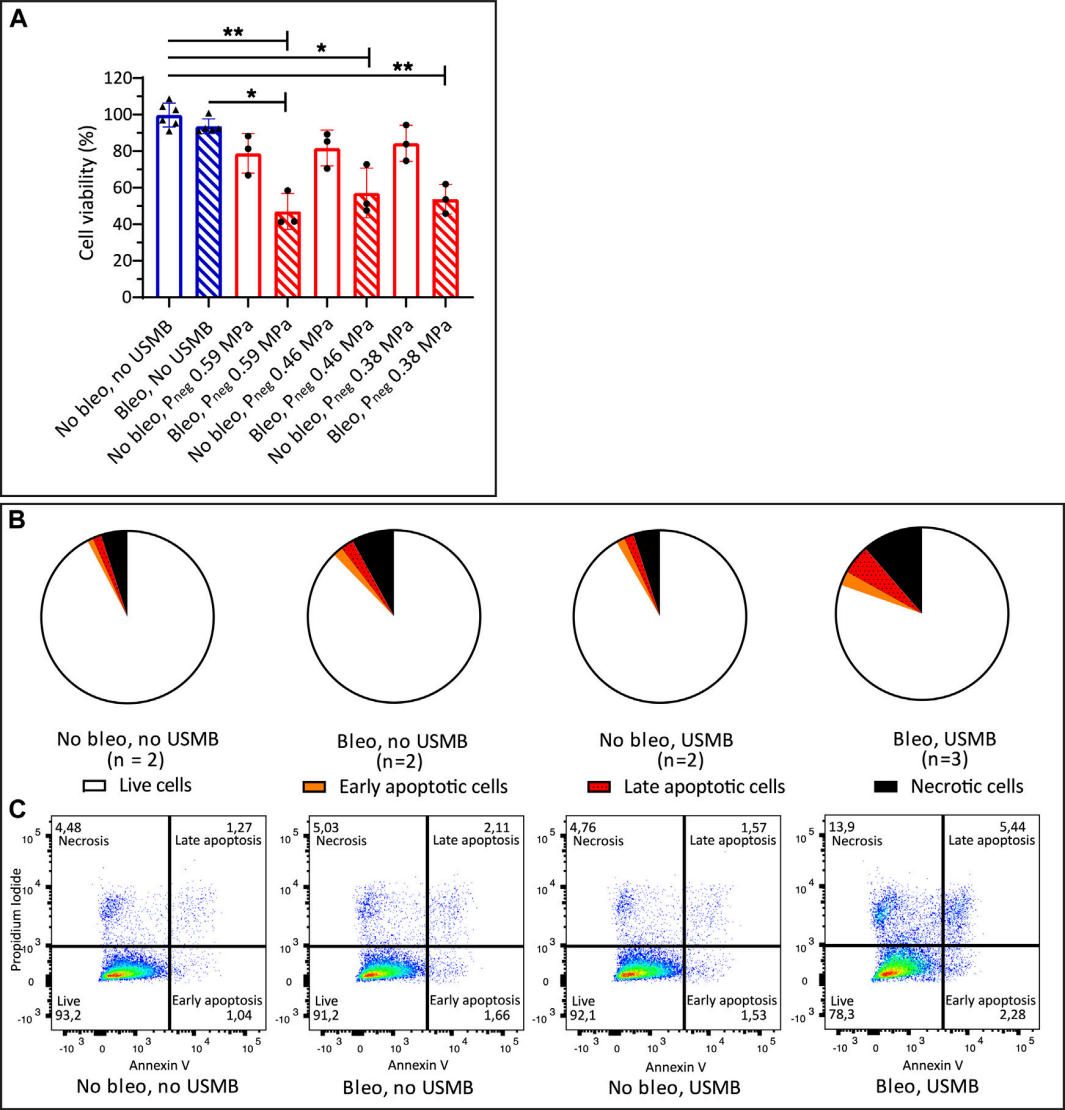
Cell viability after bleomycin ± USMB using the clinical US system with S5-1 probe **(A)** Bleomycin 10 μg/ml or NaCl 0.9% with (red, circles) and without (blue, triangles) USMB at three pressures (MI 0.6, 0.5 and 0.4). Symbols indicate individual measurements and bars indicate mean and SD (*n* ≥ 3). *p < 0.05 **p < 0.01 **(B)** Flow cytometry analysis of Propidium Iodide and Annexin V staining 48 h after bleomycin 10 μg/ml or NaCl 0.9% with or without USMB at P_neg_ 0.59 MPa (MI 0.6), pie charts represent the mean of the samples with *n* = 2 or (for bleomycin + USMB samples) n = 3. **(C)** Representative dot plots of flow cytometry analysis shown in **(B)**. More apoptosis and necrosis was observed after bleomycin + USMB. Bleo: bleomycin; MI: Mechanical Index; P_neg_: Peak negative pressure; USMB: ultrasound and microbubbles.

Figure 6 demonstrates that the cytotoxicity of bleomycin could also be increased by USMB therapy using the clinical US system with the S5-1 probe. The combination of bleomycin (10 μg/ml) and USMB with significantly decreased the cell viability compared to untreated samples at the three pressures used, while either bleomycin alone or USMB alone had little effect (Figure 6A). Addition of USMB at P_neg_ 0.59 MPa (MI 0.6) to bleomycin, also significantly decreased the cell viability from 94 to 47% compared to samples treated with bleomycin alone. At the lower pressures we also observed a decrease in cell viability when USMB was added to bleomycin (from 94 to 57% at P_neg_ 0.46 MPa and to 54% at P_neg_ 0.38 MPa), however these changes were not significant.

The results of the apoptosis assay again confirmed the decrease in cell viability with the combination of bleomycin and USMB. Figure 6B shows the mean distribution of cells over the quadrants after USMB with the clinical US system and S5-1 probe. Representative dot plots of flow cytometry analysis from experiments with two or three samples per group are shown in Figure 6C. Besides increased apoptosis, similar to what was seen with the single element transducer, also increased necrosis (11.4 vs. 8% with bleomycin alone) was observed after bleomycin plus USMB with the clinical US system.

## Discussion

In preclinical studies, USMB therapy has overcome biophysical barriers that cause heterogeneous and/or insufficient drug delivery to tumor cells, thereby increasing intracellular uptake and enhancing the efficacy of several drugs. Although the first clinical studies have been published, clinical translation of USMB therapy is still limited. We hypothesize that clinically available US systems with fixed and validated parameters will accelerate clinical translation. To pave the road forwards, we characterized several clinical probes and US-parameters and showed that effective USMB therapy can be performed *in vitro* with a non-modified clinical US system and EMA/FDA approved microbubbles.

After evaluation of three clinical US probes and a set of parameters, the US probe with the lowest center frequency (i.e., 1.6 MHz for S5-1) showed the highest USMB efficiency as measured by SG uptake. This was consistent with literature showing that a frequency close to the resonance frequency of SonoVue [i.e., 1.6–3.1 MHz depending on the bubble size (van der Meer et al., 2004)] was the most efficient (Kooiman et al., 2014; Roovers et al., 2019). Moreover, at lower pressures a larger number of cycles per pulse was beneficial. This was also seen in previous studies, although some conflicting results have been reported and intermediate pulse lengths might be optimal (Rahim et al., 2006; Karshafian et al., 2009; Phillips et al., 2010; Keller et al., 2019; Roovers et al., 2019). The SG uptake levels that we achieved with the clinical US system were comparable, or even higher depending on the transducer and US-settings, to those obtained with a custom-built US set-up with a single-element transducer and optimized US parameters (Lammertink et al., 2015a). A possible explanation for these higher uptake levels would be that the gradually increasing pressure in the Gaussian pulse shape of the PW Doppler mode leads to a more efficient bubble response than the block shaped pulse of the single-element transducer. However, we found no evidence in our microbubble attenuation experiments to support this. Since the experimental set-up and handling were equal for both set-ups and the frequencies of S5-1 probe (1.6 MHz) and the transducer of the custom-build set-up (1.5 MHz) were very similar we conclude that the improved USMB efficiency using the S5-1 and C5-1 probes must be due to other factors that we did not investigate (e.g., PRF, non-lineair US propagation and beam shape). Future experiments including cavitation measurements might further elude the underlying mechanisms.

In this study we used the PW Doppler mode for USMB therapy, in contrast to previous clinical studies that used B-mode and contrast mode, or color power angiography doppler (Clinicaltrials.gov NCT03385200, personal communication). The first clinical trial used B-mode, with settings optimized to achieve a linear acoustic signal, the maximum possible duty cycle (1%), center frequency of 1.9 MHz and MI 0.4 (measured pressure 0.27 MPa P_neg_) (Kotopoulis et al., 2013; Dimcevski et al., 2016). The second clinical trial did not provide details about specific ultrasound settings used, apart from the MI that varied between 0.4 and 1.0 (Wang et al., 2018). Although not clinically applied, PW Doppler on a clinical US system has been evaluated in a mouse study for blood-brain barrier disruption using a variation of clinically available US parameters (e.g., frequency 5.0–8.0 MHz) (Bing et al., 2009). As demonstrated by the pressure fields (Figure 2B and Supplementary Figures S1, S2), PW Doppler mode creates a very small (5.0 mm by 6.3 mm for S5-1) USMB therapy focus, much smaller than the treatment area described in the previous phase 1 clinical trial (i.e., 69 * > 100 * 1.0 mm^3^) (Kotopoulis et al., 2013). Therefore, PW Doppler mode is well suited for precisely targeted treatment. In addition, the use of a clinical US imager provides the opportunity to perform imaging and therapy consecutively, thus performing image-guided therapy.

This is to our best knowledge the first *in vitro* study that evaluates the effect of USMB therapy using a clinical US system and approved SonoVue microbubbles, while performing extensive evaluation of multiple transducers and US settings available in PW Doppler mode. Previously, *in vitro* studies have used Optison microbubbles for USMB therapy with clinical US systems in spectral Doppler, 2-D scan mode or harmonic imaging (Octave) mode at a frequency of 1.5 of 3.5 MHz (Miller and Quddus, 2000; Miller et al., 2003). Compared to our findings, these methods resulted in a lower USMB efficacy (below 10%), which could indicate that PW Doppler mode is more effective. Other *in vitro* studies have used a diagnostic US system to evaluate microbubble response, while therapeutic USMB was omitted or administered with a non-clinical transducer (Keravnou et al., 2015; Keller et al., 2019; Keller et al., 2021). USMB therapy would benefit from simultaneous (real-time) cavitation monitoring with a single transducer of a clinical US system. This solution would allow for monitoring of bioeffects (Chen et al., 2003; Hallow et al., 2006; Tamosiunas et al., 2012; Maciulevicius et al., 2015), while using standardized US settings. Currently, simultaneous USMB therapy and cavitation monitoring is not yet available on clinical US systems, although our work and the work by Keller et al. show it is technically feasible (Keller et al., 2021). Meanwhile, our approach leads to a standardization of US parameters used and may be immediately used in clinics.

Next to correct determination and extensive reporting of the US exposure conditions used (ter Haar et al., 2011), which has been performed for clinical US systems, and performing cavitation monitoring during treatment, the use of mono-disperse microbubbles will further reduce the disparity of experimental results. Currently, commercial, clinically approved microbubbles are polydisperse. However, recent papers show that monodisperse microbubbles have a more uniform acoustic response and an increased imaging sensitivity (Segers et al., 2018; Helbert et al., 2020), which will also improve the reproducibility and controllability of USMB therapy.

We hypothesized that USMB therapy with a clinical US system and approved microbubbles could improve the local efficacy of chemotherapy. Both the Alamar Blue assay and the flow cytometry analysis showed that *in vitro* USMB therapy with both the clinical and the custom-built US system clearly increased the cytotoxicity of the hydrophilic drug bleomycin. However, our absolute IC_50_ values have to be interpreted with caution. The nonlinear regression model included only one concentration above the IC_50_ for the cells treated with USMB, and none for the cells treated without USMB. This led to a large confidence interval in the IC_50_ estimations. Unfortunately, due to a worldwide shortage of bleomycin (Carrai, 2019) it was not feasible to increase the concentration further, in order to achieve an effect closer to 100% cell death. For this reason, we additionally calculated the IC_25_ of each group and compared those. These data confirmed the increased cytotoxicity of bleomycin with the addition of USMB therapy.

While in clinical practice bleomycin is only used in a few tumor types, these results could be extended to a wide range of treatments with other hydrophilic chemotherapeutics. For example, based on previous *in vitro* results of Lammertink et al. future patients receiving chemotherapy or chemoradiation containing cisplatin could benefit from the addition of local USMB therapy (Lammertink et al., 2016). Furthermore, USMB therapy could be used to enhance the effect of therapeutic antibodies or nanoparticles (Heath et al., 2012; Togtema et al., 2012; Bellary et al., 2020; Snipstad et al., 2021a; Snipstad et al., 2021b). Finally, clinical studies evaluating the potential of USMB therapy in addition to radiotherapy in the absence of drugs are ongoing (Shi et al., 2021) (Clinicaltrials.gov NCT04431674, NCT04431648).

The custom-made TwentiCells used in our experiments are an attractive alternative to for example CLINIcells and provide the opportunity to perform USMB experiments with a large number of independently sonicated samples, while using small volumes of medium, drugs and microbubbles. In addition, the TwentiCells hardly interfere with the applied ultrasound field, a common limitation of *in vitro* US set-ups (Hensel et al., 2011; Leskinen and Hynynen, 2012). To obtain reliable and reproducible results we standardized the procedures throughout our experiments as much as possible. This is essential, as many parameters [e.g., position of cells with respect to transducer, time between preparation and use of microbubbles, time between addition of microbubbles and sonication (Keller et al., 2019; Beekers et al., 2020)] can affect outcome of USMB therapy.

To conclude, we have shown that a non-modified clinical US system in combination with clinically approved microbubbles can be used to perform highly effective USMB therapy *in vitro*. The next step towards clinical translation is to apply these methods *in vivo*. Future trials should determine the safety and efficacy of our methods and US parameters in patients.

## Data Availability

The raw data supporting the conclusion of this article will be made available by the authors, without undue reservation.

## Footnotes

1
https://pubchem.ncbi.nlm.nih.gov/compound/Bleomycin#section=CAS [Accessed August 31st, 2021].
