# Supplementary material for: Ultrasound-Mediated Drug Delivery With a Clinical Ultrasound System: In Vitro Evaluation
Source: Front Pharmacol. 2021 Oct 19;12:768436. doi: 10.3389/fphar.2021.768436 (PMC8560689; doi:10.3389/fphar.2021.768436)
Supplement: Supplementary file 1 [file DataSheet1.PDF]

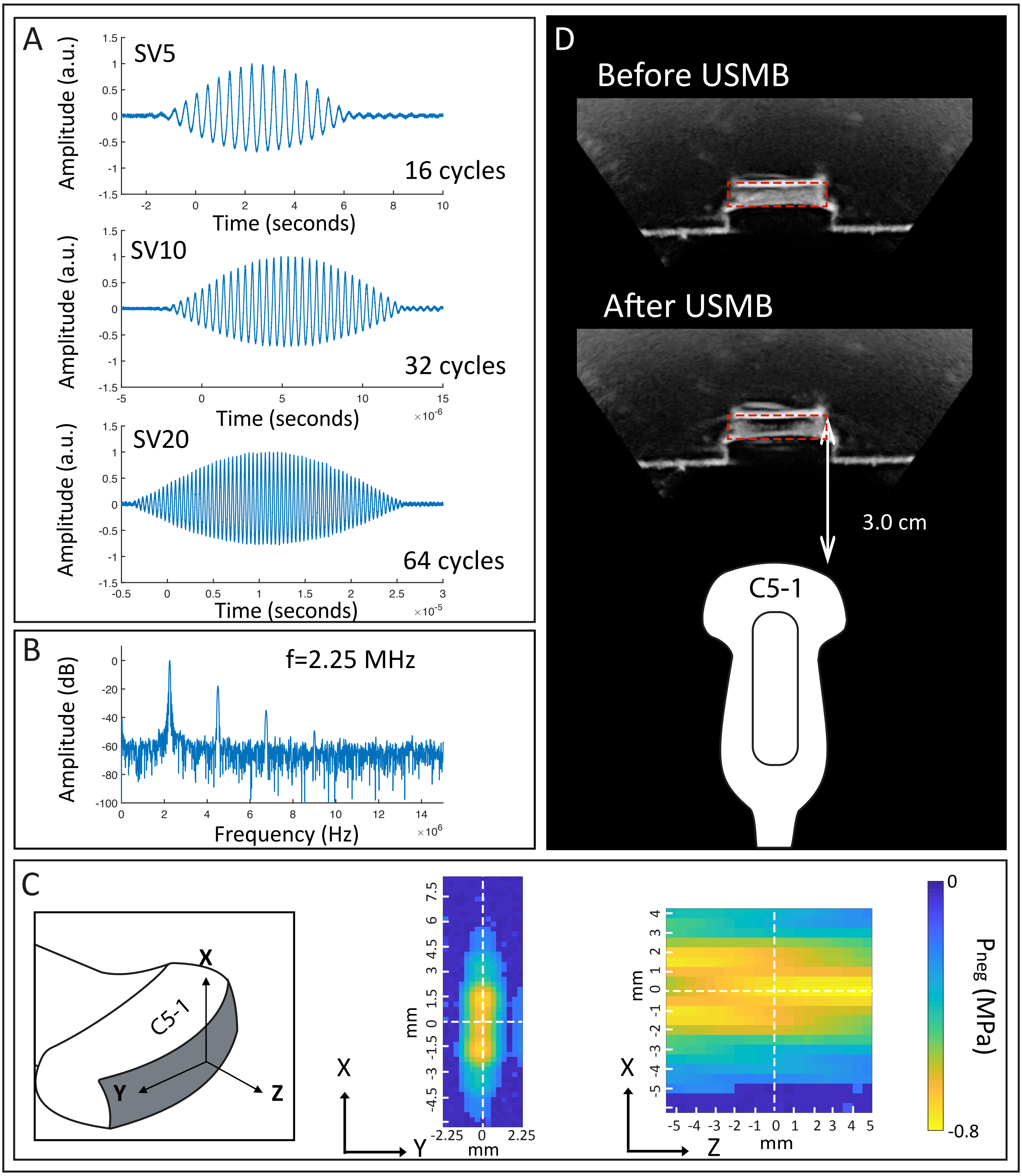


Supplementary figure 1. Characteristics of C5-1 probe **(A)** Shape of emitted ultrasound pulses in PW mode for different pulse length. **(B)** Frequency spectrum of SV 20mm pulse. **(C)** Pressure field maps in PW mode. **(D)** B-mode images of Twenti-Cell (red rectangle) containing microbubbles, before and after USMB therapy (15 seconds at MI 0.8, SV 20mm).

SV: sample volume; f: frequency; a.u.: arbitray units; $P_{neg}$: Peak negative pressure.

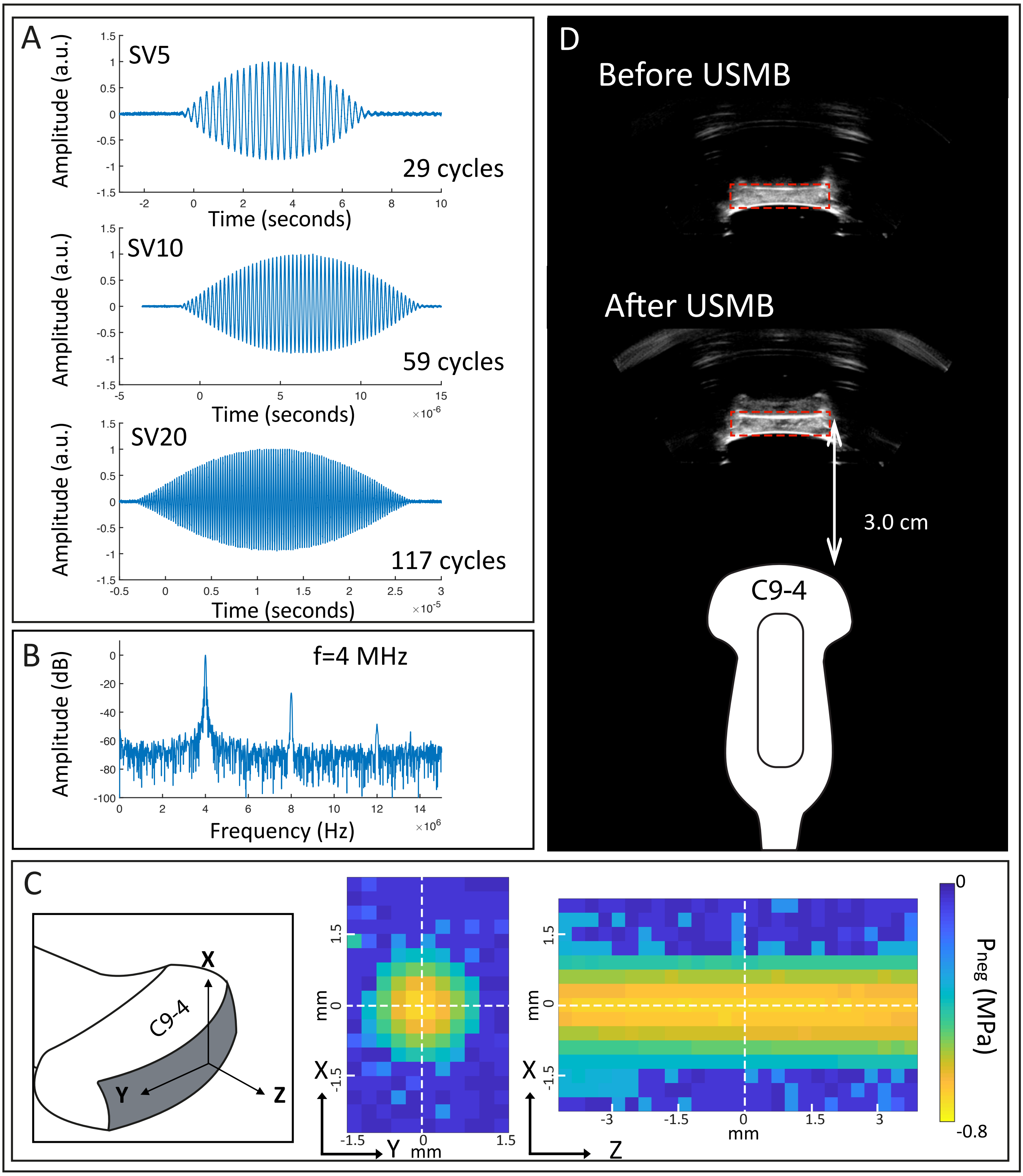


Supplementary figure 2. Characteristics of C9-4 probe **(A)** Shape of emitted ultrasound pulses in PW mode for different pulse length. **(B)** Frequency spectrum of SV 20mm pulse. **(C)** Pressure field maps in PW mode. **(D)** B-mode images of Twenti-Cell (red rectangle) containing microbubbles, before and after USMB therapy (15 seconds at MI 0.3, SV 20mm).
SV: sample volume; f: frequency; a.u.: arbitray units; $P_{neg}$: Peak negative pressure.

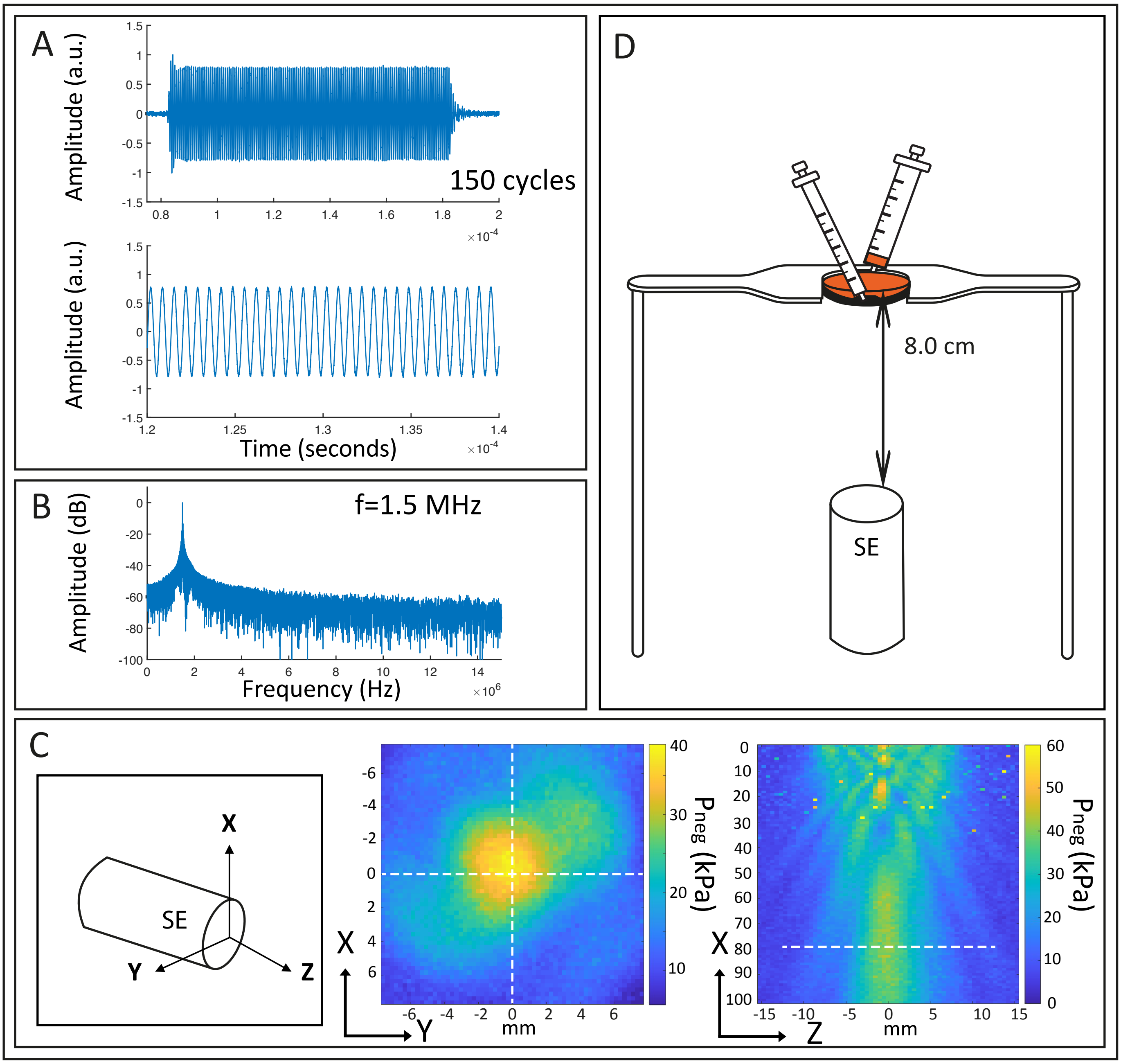


Supplementary figure 3. Characteristics of custom-build US set-up with single-element transducer **(A)** Shape of emitted ultrasound pulses. **(B)** Frequency spectrum. **(C)** Pressure field maps. Left: transversal plane. Right: axial plane, white line indicates position of transversal plane. **(D)** Experimental set-up. f: frequency; a.u.: arbitray units; SE: single element transducer.
